# The Antibacterial Properties of a Reinforced Zinc Oxide Eugenol Combined with Cloisite 5A Nanoclay: An In-Vitro Study

**DOI:** 10.3390/jfb15070198

**Published:** 2024-07-20

**Authors:** Bahareh Nazemisalman, Shaghayegh Niaz, Shayan Darvish, Ayda Notash, Ali Ramazani, Ionut Luchian

**Affiliations:** 1 Department of Pediatric Dentistry, School of Dentistry, Zanjan University of Medical Sciences, Zanjan 4513956184, Iran; drnazemi@zums.ac.ir; 2 Independent Researcher, Urmia 5719175546, Iran; dr.shaghayeghniyazi1999@gmail.com; 3 School of Dentistry, University of Michigan, Ann Arbor, MI 48104, USA; 4 Independent Researcher, Tabriz 5178654714, Iran; aidantsh78@gmail.com; 5 Department of Pharmaceutical Biotechnology, School of Pharmacy, Zanjan University of Medical Sciences, Zanjan 4513956184, Iran; 6 Department of Periodontology, Faculty of Dental Medicine, “Grigore T. Popa” University of Medicine and Pharmacy, 700115 Iasi, Romania; ionut.luchian@umfiasi.ro

**Keywords:** zinc oxide eugenol, nanoclay, antibacterial agents, nanoparticles, *Streptococcus mutans*, *Escherichia coli*, *Enterococcus faecalis*

## Abstract

Pulpotomies and pulpectomies are the most common clinical approach for dental caries in the primary dentition. Reinforced zinc oxide eugenol (ZOE) is an ideal material for filling in the pulp chamber after pulp therapies. The aim of this study was to assess the addition of Cloisite 5A nanoclay material to ZOE and evaluate its antibacterial properties. In this case–control study, the nanoclay nanoparticles were dissolved using a solvent (Eugenol) in different concentrations and their antibacterial properties were assessed using the agar diffusion test and biofilm analysis of *Streptococcus mutans* (*S. mutans*), *Enterococcus faecalis* (*E. faecalis*), and *Escherichia coli* (*E. coli*) in in vitro conditions using the AATCC 100 standards. The diameter of the inhibition zone was measured and assessed statistically using the SPSS software (Version 28, IBM, Chicago, IL, USA) with a significance level of 0.05. The antibacterial properties of the ZOE with nanoclay particles were significantly greater in comparison to the plain ZOE against *E. faecalis*, *S. mutans*, and *E. coli*. The inhibition zone against *E. coli* under the effect of the ZOE and nanoclay particles combined was significantly higher than that against *E. faecalis* and *S. mutans*. The current study showed that the addition of Cloisite 5A nanoclay particles can improve the antibacterial properties of ZOE significantly at certain concentrations.

## 1. Introduction

Dental caries is a public health concern worldwide which is affecting children at an early age, resulting in adverse effects on their oral and general health status [1]. When caries reaches the pulp, pulp therapies such as pulpotomies and pulpectomies are amongst the first-choice treatments [2]. If left untreated, these pulpal infections can lead to painful experiences for children resulting in nutritional deficiencies, esthetic concerns, and general growth deficiencies [3]. Therefore, it is essential to save the primary dentition until the exfoliation and eruption of the permanent dentition. If the pulpal chamber becomes exposed to the oral cavity, pulpal therapy is needed for the primary dentition [4,5]. Pulpotomy is one of the most common pulp therapies if the pulp is diagnosed with asymptomatic reversible pulpitis. The success rate depends on multiple factors, including, but not limited to, a correct pulpal diagnosis, pulpal bleeding, and the placement of an ideal cement which will be in direct contact with the remaining pulpal tissue in the canals [6,7]. If a pulpotomy treatment is not feasible, a pulpectomy or extraction of the primary dentition are the next available options. However, it is clinically recommended to preserve the primary dentition [8]. In a pulpectomy, after the cleaning and shaping of the root canals, a cement should be placed in the canals that is antibacterial, provides a good seal, is radiopaque, but is washed away if extruded from the apical foramen [3]. Different cements and materials are available for this purpose [9]. Amongst them, reinforced zinc oxide eugenol (ZOE) is a polymer that has been reinforced with a 20–40% Poly methyl methacrylate (PMMA) [10]. ZOE has sedative and antibacterial properties [11,12]. Moreover, it will not damage the peri radicular area in case excess material exiting the root canals and the excess is absorbed, which makes it a suitable material for pulpal treatments such as pulpotomy and pulpectomies that has been used since 1930 [13,14]. Since the primary dentition roots are absorbed in the exfoliation process, ZOE, with a reported success rate of 78.5% [15], as the most accessible and affordable material worldwide, continues to be a clinically acceptable material and is commonly used for pulpectomies [16,17]. However, ZOE’s antibacterial properties are not ideal [18,19] and its resorption rate is slower when compared to that of the tooth root [20].

Dental materials are improving every day in terms of their antimicrobial properties and biocompatibility [21]. Along the same lines, the addition of nanoparticles to dental materials such as composites has recently shown significant improvements in their mechanical and antibacterial properties [22,23,24,25,26]. Nanoparticles have a small size but a remarkable surface area to interact with bacteria and affect them [27]. Cloisite nanoclay montmorillonites (MMTs) have shown antibacterial effects in previous research [28]. They are nanoclays that are octagonal and are placed in between layers of tetra-silicate, which has been used in polymers to improve their mechanical properties [24,29,30,31]. The use of MMT nanoclays such as Cloisite 5A and 20A in dental materials has been shown to improve their mechanical and adhesion properties at concentrations of 0.5 to 5% [32,33,34]. Nanoclay particles have been shown to have antibacterial properties [25]. Moreover, they are not toxic to the human body and have shown antibacterial effects even at a low weight percentage in materials without any adverse effects [35]. Ghorbanpour et al. have shown in their study that the addition of nanoclays to silver materials significantly increased their antibacterial effect [36].

*Enterococcus faecalis (E. faecalis)* is a part of the oral microbiome and has been recognized as one of the main bacteria in primary root canal infections. Moreover, it has been identified in chronic periapical periodontitis tissues [37]. *Streptococcus mutans (S. mutans)* is the main cariogenic bacteria and a part of the oral microbiome. It can damage the enamel by producing lactic acid and is one of the main bacteria behind dental pain and dental abscesses in the primary dentition [38,39]. *Escherichia coli (E. coli*) is a part of the oral and digestive system microbiome. It is usually harmless but can cause diarrhea and respiratory or urinary tract infections [40,41].

In addition to the mechanical, antibacterial, and biocompatibility benefits mentioned above, nanoclay is a material that is readily available, accessible, inexpensive, has good thermal stability, and can be easily mixed with dental materials [35,42]. Thus, this study was designed to investigate the antibacterial effect of nanoclays in addition to reinforced ZOE in comparison to flat reinforced ZOE against *S. mutans*, *E. faecalis*, and *E. coli* in an in vitro environment to improve ZOE’s antibacterial properties, as the most commonly used material for pulpectomies.

## 2. Materials and Methods

This case–control study consisted of 6 different groups:

1. Zonalin 1 (Z1): 100 wt% zonalin (negative control).
2. Zonalin 2 (Z2): 80 wt% zonalin + 20 wt% nanoclay.
3. Zonalin 3 (Z3): 60 wt% zonalin + 40 wt% nanoclay.
4. Zonalin 4 (Z4): 40 wt% zonalin + 60 wt% nanoclay.
5. Zonalin 5 (Z5): 20 wt% zonalin + 80 wt% nanoclay.
6. Zonalin 6 (Z6): 100 wt% nanoclay (positive control).

Three antibacterial tests were used to assess the materials in the above groups:

1. The disk diffusion test by measuring the diameter of inhibition zone.
2. The well diffusion test by measuring the diameter of inhibition zone.
3. The microtiter dish assay.

### 2.1. Material and Bacteria Preparation

Zonalin (Golchai, Karaj, Alborz, Iran) consisted of 30 mg of zinc oxide and 18 mL of eugenol. Nanoclays were made of Cloisite 5A and had an average size of 95 nm (Southern Clay Products Inc., Gonzales, TX, USA). Nanoclay powder and zinc oxide powder weight were measured using a digital scale (Ohaus SP601 scout Pro, Parsippany, NJ, USA), and mixed on a vibrator until visually well dispersed. Later, the combination powder was mixed with eugenol using a sterile metallic mixing spatula to mimic the actual clinical method used for pulpectomies in the pediatric population.

The bacteria, *S. mutans* (PTCC 1683),* E. faecalis* (ATCC 29212), and *E. coli* (ATCC 25922), were collected from a microbiology lab (Zanjan Universirty of Medical Sciences, Zanjan, Iran) in lyophilized format. Broth, blood agar, and Muller–Hinton agar were used for antibacterial tests and for bacteria growth. The culture plates were prepared using the manufacturers manual (Merck, Darmstadt, Germany). Suspensions were prepared from the bacteria, and they were transferred to Muller–Hinton agar culture plates, except for *S. mutans,* which was transferred to blood agar plates. They were placed in an incubator (37 °C) for bacteria to multiply. All the bacteria samples were diluted using BHI broth to the 0.5 McFarland standard.

### 2.2. Disk Diffusion Test: (n = 9)

Antibiogram disks were prepared with a 6 mm diameter and soaked in the materials mixed with Zonalin and nanoclay (Z1 to Z6). Blood agar plates of 10 cm diameter were used for *S. mutans*, and Muller–Hinton agar was used for *E. faecalis* and *E. coli.* A volume of 200 µL of the 0.5 McFarland bacteria sample was placed on the appropriate plates and diffused using a sterile swab. The disks containing Z1 to Z6 were placed on the plates with a negative control disk containing no material in the center of the plates. The plates were placed in an incubator (37 °C) for 24 h. The plates containing *S. mutans* samples were placed in a CO_2_ incubator (37 °C, 24 h) to grow in an anaerobic environment. The zone of inhibition around the disks was measured using a caliper in mm. This test was repeated for each bacterial species separately.

### 2.3. Well Diffusion Test: (n = 9)

A volume of 200 µL of the 0.5 McFarland bacteria sample was placed on the appropriate plates (blood agar for *S. mutans* and Muller–Hinton agar for *E. faecalis* and *E. coli)* and diffused using a sterile swab. Six wells were formed on the plates with 5 mm diameter within 2 cm of each other. A total of 0.15 g of each of the combinations (Z1–Z6) was placed in each well. All the plates were placed in an incubator (37 °C) for 24 h. *S. mutans* samples were placed in a CO_2_ incubator to grow in an anaerobic environment. The zone of inhibition around the disks was measured using a caliper in mm. This test was repeated for each bacterial species separately.

### 2.4. The Microtiter Dish Assay: (n = 9)

**Figure 1:**
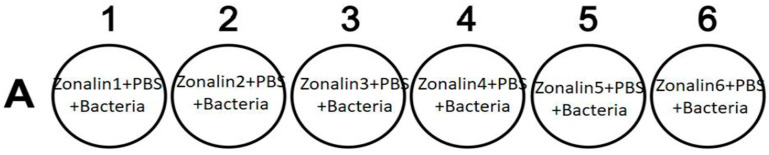
The 24-well map for microtiter dish assay (Z1 to Z6).

A total of 0.2 g of each of the combinations (Z1–Z6) was placed in a glass vial with 2 mL of phosphate-buffered saline (PBS). Vials were vortexed and placed in a shaker incubator for 24 h (37 °C, 81 rpm). The extracts were collected and placed in a microtube. A volume of 150 µL of the extracts was placed in a 24-well plate including one control well of only 150 µL of PBS. The map of the 24-well plate is shown in Figure 1.

In each well, 50 µL of 0.5 McFarland bacteria suspension was added. This test was repeated for each bacterial species separately. The plates were placed in an incubator (37 °C) for 24 h. The plates containing *S. mutans* samples were placed in a CO_2_ incubator to grow in an anaerobic environment. After incubation, the plates were emptied using a sampler and washed with 200 µL of PBS 3 times to remove the unattached bacteria. A volume of 200 µL of a 4% crystal violet solution was added to the wells to stain the attached bacteria. After 12 min, the stain solution was removed and washed with distilled water. A volume of 200 µL of 33% acetic acid was added to each well to release the color attached to the bacteria for the ELISA plate reader 15 min prior to the reading. The plates were then read at 600 nm with the plate reader (Tecan EL-Reader, Männedorf, Switzerland).

The results were statistically analyzed using the Shapiro–Wilk and one-way ANOVA tests by SPSS. Methods and materials for this study were approved by the ethics committee of Zanjan University of Medical Sciences (IR.ZUMS.BLC.1401.001).

## 3. Results

### 3.1. Disk Diffusion Test

**Figure 2:**
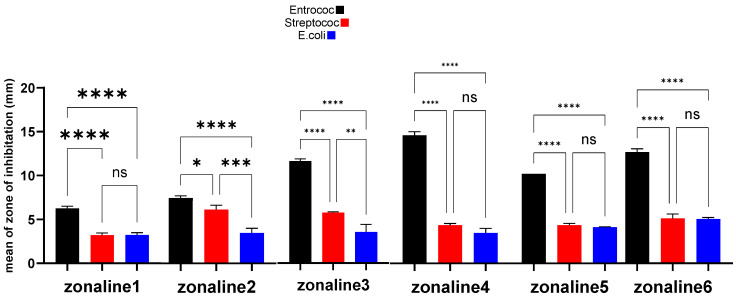
Comparison of inhibition zones in the disk diffusion test. ns: not significant, *: *p*-value < 0.05, **: *p*-value < 0.01, ***: *p*-value < 0.001, ****: *p*-value < 0.0001.

The results are presented in Figure 2. The results for each bacterial species are presented separately in Table 1.

### 3.2. Well Diffusion Test

**Figure 3:**
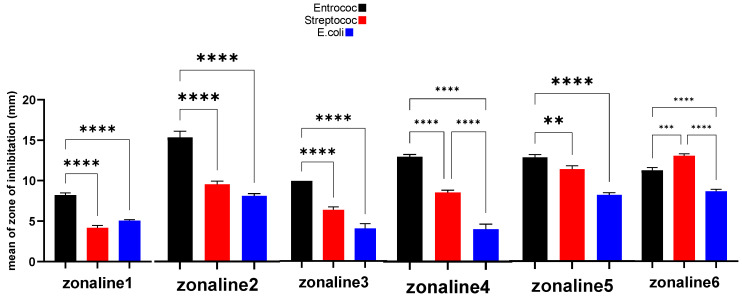
Comparison of inhibition zones in the well diffusion test. **: *p*-value < 0.01, ***: *p*-value < 0.001, ****: *p*-value < 0.0001.

**Figure 4:**
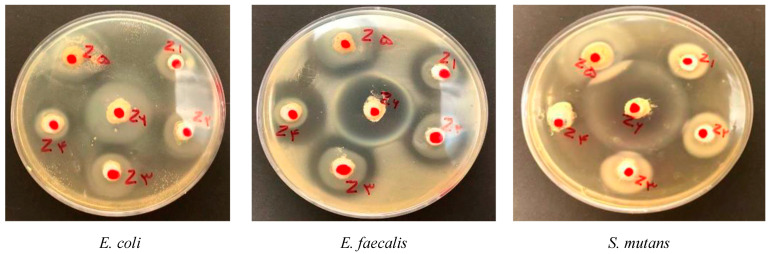
Inhibition zones for well diffusion test for each bacterial species.

The results are presented in Figure 3. The results for each bacterial species are presented separately in Table 2. A sample picture of the well diffusion test is presented in Figure 4.

### 3.3. Microtiter Dish Assay

**Figure 5:**
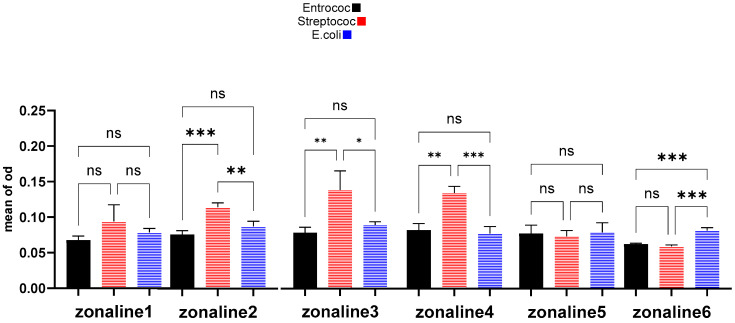
Comparison of Z1 to Z6 in microtiter dish assay test. ns: not significant, *: *p*-value < 0.05, **: *p*-value < 0.01, ***: *p*-value < 0.001.

The results are presented in Figure 5. The results for each bacterial species are presented separately in Table 3.

## 4. Discussion

We showed that the addition of Cloisite 5A nanoclay particles to Zonalin can enhance its antibacterial properties. The material weight percentage with the greatest antibacterial effect on each bacterial species will be discussed below.

### 4.1. E. faecalis

In the well diffusion method, the Z5 group (20 wt% zonalin + 80 wt% nanoclay), and in the disk diffusion method, the Z4 group (40 wt% zonalin + 60 wt% nanoclay) showed the greatest antibacterial effect. This is in line with the study that Airis et al. conducted in 2020. They showed that the addition of silver nanoparticles can interfere with bacterial signaling [43]. Bacterial signaling is a process conducted by protein phosphorylation [44]. The nanoclay particles will cause tyrosine dephosphorylation and thus interrupt bacterial signaling, leading to the apoptosis of the cell.

### 4.2. S. mutans

In the well diffusion method, the Z5 group (20 wt% zonalin + 80 wt% nanoclay), and in the disk diffusion method, the Z3 group (60 wt% zonalin + 40 wt% nanoclay) showed the greatest antibacterial effect. This is in contrast with the results of Barzegar et al. They showed in their study that the addition of Cloisite 20A nanoclay did not improve the antibacterial and mechanical properties of PMMA [45]. The reason behind this may be the fact that they used the 20A nanoclay in their study and we used the 5A nanoclay. At the nanoscale, the size and morphology of the nanoparticles can significantly affect their antibacterial properties [46]. The 5A Cloisite nanoclay has a size below 100 nm. Smaller particles, and specifically those below 100 nm, can infiltrate the cell membranes more efficiently in comparison to larger particles and thus can interact with proteins and interrupt DNA synthesis [47,48].

### 4.3. E. coli

In the well diffusion method, as well as in the disk diffusion method, the Z5 group (20 wt% zonalin + 80 wt% nanoclay) showed the greatest antibacterial effect. This is in line with the study that Ghorbanpour et al. conducted. They showed that the addition of montmorillonite K10 nanoclay particles can enhance the antibacterial properties in combination with silver nanoparticles [36]. The antibacterial effect of the mixture increased by increasing the nanoclay particle concentration in the mixture, from group Z1 to Z6.

In general, the antibacterial mechanism of nanoclay particles can be explained through their interactions with the bacteria cell wall and membrane leading to cell lysis, interactions with proteins and protein synthesis interruption, and interactions with bacteria DNA and preventing DNA replication [49]. Amongst the bacteria studied, *E. coli* showed the greatest sensitivity under the effect of the nanoclay particles. That is to say, going from group Z1 to Z5, the antibacterial effect against *E. coli* increased significantly. The 80% nanoclay group showed the greatest antibacterial effect. The reason behind *E. coli* sensitivity could be that it is a Gram-negative bacteria while *S. mutans* and *E. faecalis* are both Gram-positive bacteria. The Gram-positive bacteria have a thick cell wall consisting of peptidoglycan and lipopolysaccharides averaging from 20 to 80 nm, which makes them more resistant to the penetration of antibacterial agents. A previous study has shown that the Gram-positive bacteria have a thick cell wall which can prevent the nanoclay particles from infiltrating their cells [50,51].

The microtiter dish assay results showed antibacterial effects, although they were not consistent and did not present a pattern in the antibacterial properties. An explanation can be the hydrophobic dissolvent of the mixtures (Z1 to Z6) in the PBS. The materials contained Eugenol, which is a hydrophobic material [52]. PBS is also a hydrophilic material, and thus the dissolvement might have been interrupted. Thosar et al. have investigated the dissolvement of different materials including eugenol. Eugenol displayed the least solubility amongst the materials studied, and thus, this could be a reason behind not observing a consistent pattern in the microtiter dish assay [53]. Amongst the antibacterial tests performed in this study, the well diffusion method showed the most promising results and was the most reliable method for studying dental materials combined with nanoparticles in comparison to the disk diffusion and the microtiter dish assay.

This is the first study that has investigated the antibacterial effects of nanoclays in combination with reinforced ZOE (Zonalin) in an in vitro study. Nanoparticles affect bacteria through damaging the bacteria cell wall, causing DNA damage and electron transport damage, and interfering with sulfhydryl protein synthesis and causing oxidative damage [47,48]. Cloisite nanoclays mostly act through cell penetration and causing a rupture in bacteria, leading to lysis [54]. However, other mechanisms such as those acting through quaternary ammonium salts in their compositions have also been reported [55]. We chose a nanoclay size of below 100 nm to increase the chance of bacteria cell membrane penetration and increase the associated antibacterial effects. A study by Barzegar et al. has shown that the use of large nanoclay particles (cloisite 20A) can work against the mechanical properties of PMMAs [45]. We have shown improved antibacterial activity in the ZOE cement when combined with cloisite 5A nanoclay in in vitro conditions. Due to the limitations of this study, we were not able to conduct an SEM analysis; however, we recommend that future studies focus on investigating the effect of nanoclays in combination with other dental materials and on a combination of oral microflora to resemble in vivo conditions more accurately. Additionally, the cytotoxicity, microscopic analysis, and mechanical properties of the of the above combinations should be investigated to assure their safe use in a clinical setting.

## 5. Conclusions

We showed that the addition of Cloisite 5A nanoclay particles enhanced the antibacterial effects of reinforced ZOE. These particles can also be used in toothpaste, mouthwash, endodontic sealers, or in different dental materials such as resins. The nanoclay particles can be used to replace the chemical materials currently used for pulpotomies and pulpectomies in the primary dentition. Further studies are needed, however, to investigate the cytotoxicity and antibacterial properties of the nanoclays in in vivo conditions.

## Figures and Tables

**Table 1:** Inhibition zone hierarchy for disk diffusion test for each bacterial species.

Bacterial Species	Zonalin Group					
*E. coli*	Z6>	Z5>	Z3>	Z4 = Z2>		Z1
*S. mutans*	Z3>	Z2>	Z6>	Z5 = Z4>		Z1
*E. faecalis*	Z4>	Z6>	Z3>	Z5>	Z2>	Z1

Zonalin groups are listed based on inhibition zones from largest to smallest (left to right).

**Table 2:** Inhibition zone hierarchy for well diffusion test for each bacteria.

Bacterial Species	Zonalin Group					
*E. coli*	Z6>	Z5>	Z1>	Z2>	Z3>	Z4
*S. mutans*	Z6>	Z5>	Z4>	Z3>	Z2>	Z1
*E. faecalis*	Z6>	Z5>	Z4>	Z3 = Z2>		Z1

Zonalin groups are listed based on inhibition zones from largest to smallest (left to right).

**Table 3:** Antibacterial effects for the microtiter dish assay for each bacterial species.

Bacterial Species	Zonalin Group					
*E. coli*	Z4>	Z1>	Z5>	Z6>	Z2>	Z3
*S. mutans*	Z6>	Z5>	Z1>	Z2>	Z4>	Z3
*E. faecalis*	Z6>	Z1>	Z2>	Z5>	Z3>	Z4

Zonalin groups are listed based on OD readings from largest to smallest (left to right).

## Data Availability

The raw data supporting the conclusions of this article will be made available on reasonable request by contacting Dr. Bahareh Nazemisalman at drnazemi@zums.ac.ir.
